# Selected Interleukins Relevant to Multiple Sclerosis: New Directions, Potential Targets and Therapeutic Perspectives

**DOI:** 10.3390/ijms252010931

**Published:** 2024-10-11

**Authors:** Hubert Mado, Artur Stasiniewicz, Monika Adamczyk-Sowa, Paweł Sowa

**Affiliations:** 1 Department of Neurology, Faculty of Medical Sciences in Zabrze, Medical University of Silesia in Katowice, ul. 3 Maja 13/15, 41-800 Zabrze, Poland; 2 Department of Otorhinolaryngology and Oncological Laryngology, Faculty of Medical Sciences in Zabrze, Medical University of Silesia in Katowice, 40-055 Katowice, Poland

**Keywords:** multiple sclerosis, MS, interleukins, ILs, microglia, neuroinflammation, neuroprotection, cytokines

## Abstract

Multiple sclerosis (MS) is a chronic inflammatory disease of the central nervous system (CNS) that progresses with demyelination and neurodegeneration. To date, many studies have revealed the key role of interleukins in the pathogenesis of MS, but their impact has not been fully explained. The aim of the present study was to collect and review the results obtained so far regarding the influence of interleukins on the development and course of MS and to assess the potential for their further use. Through the platform “PubMed”, terms related to interleukins and MS were searched. The following interval was set as the time criterion: 2014–2024. A total of 12,731 articles were found, and 100 papers were subsequently used. Cells that produce IL-10 have a neuroprotective effect, whereas those that synthesize IL-6 most likely exacerbate neuroinflammation. IL-12, IL-23 and IL-18 represent pro-inflammatory cytokines. It was found that treatment with an anti-IL-12p40 monoclonal antibody in a study group of MS patients showed a beneficial effect. IL-4 is a pleiotropic cytokine that plays a significant role in type 2 immune responses and inhibits MS progression. IL-13 is an anti-inflammatory cytokine through which the processes of oligodendrogenesis and remyelination occur more efficiently. The group of interleukins discussed in our paper may represent a promising starting point for further research aimed at finding new therapies and prognostic markers for MS.

## 1. Introduction 

Multiple sclerosis (MS) is a chronic inflammatory disease of the central nervous system (CNS) with progressive demyelination of neurons and neurodegeneration [1]. The disease is the leading cause of disability in young adults due to neurological causes [2,3]. The current dominant concept that seems to explain the onset and course of MS is the view of its autoimmune basis. So far, high-efficacy therapeutic approaches have been introduced [4]. Treatment focuses primarily on reducing the number of relapses, as well as minimizing the progression of the disease, including physical disability and cognitive impairment [5]. However, it is necessary to continue the search for even more effective and optimal methods in the treatment of MS. 

Many studies conducted to date have revealed the key role of interleukins in the pathogenesis of MS, but their influence on the onset and progression of the disease has not been fully explained [2]. 

The autoimmune process, regulated by cytokines, is a factor leading to the progression of the disease and the formation of MS relapses. Cytokines have an important function in this process [5]. It has so far been shown that in the active phase of MS, the levels of interleukin 2 (IL-2), IL-3, IL-4, IL-6, IL-13, IL-17, IL-21, IL-22 and IL-33, among others, are elevated, compared to a control group of healthy individuals and patients in remission. IL-10, on the other hand, has shown an effect in preventing disease progression [6]. 

The results of some studies published to date have shown neuroprotective effects of IL-13, IL-22 and IL-33. Recent studies have emphasized the important role of microglia-associated interleukins in the pathogenesis of MS [7]. Among the interleukins with positive effects in the course of MS, we can include IL-4, IL-10, and IL-13, while those with negative effects include interleukin 1β (IL-1β), IL-6, IL-18, IL-12, and IL-23.

An invaluable contribution to the development of knowledge about the pathomechanism and dynamics of MS development in humans, as well as the assessment of the possibility of therapeutic applications, should be attributed to animal models such as experimental autoimmune encephalomyelitis (EAEs) [8]. An important advantage of studying these models is the fact that material from the human CNS is difficult to collect, and often even impossible to obtain. Currently, genetically modified mouse models, including humanized ones, are used, which results in a wide range of possibilities for conducting innovative research [9]. Particular attention is paid to models created in such a way that the adaptive pool of both T and B lymphocytes is directed to auto-aggressive molecules, characteristic of the myelin protein. However, it should be emphasized that T lymphocytes mature and are activated in vitro, and they are artificially modified; therefore, their morphology and mode of action are not entirely consistent with T lymphocytes formed in vivo [8]. Targeting research to understand the nature of the influence of interleukins on the course and development of multiple sclerosis would seem to provide support for finding new therapeutic approaches. However, more in-depth research is still needed to better understand the nature of their action and impact. 

This review aims to discuss and summarize the results obtained so far regarding the importance of IL in the pathogenesis of MS, as well as to assess the possibility of using these molecules as predictors, as well as their use in diagnosis and potentially in the therapeutic sphere.

## 2. Methods 

Using “PubMed,” the following terms were searched: “microglia”, “microglia cells”, “interleukins in MS”, IL-1-beta, IL-6, IL-18, IL-12, IL-23, IL-4, IL-10, and IL-13. The time criterion for the searched publications was set from 2014 to 2024, inclusive. Some publications were searched beyond this timeframe. The results of studies conducted in all populations were taken into account. The vast majority of articles were written in English. The search for publications was performed between 1 April and 30 June 2024. Both abstracts and full versions of articles were considered. The search considered all types of manuscripts, including review papers, original papers and case reports.

While reviewing PubMed for relevant papers, work was already underway on this article. A total of 12,731 articles were found. Duplicate and irrelevant papers were omitted. Ultimately, 100 publications were used. After the search was technically completed, a review of the related literature continued until 30 June 2024, as well as monitoring of the validity of the data used. Articles cited in other reviews on similar topics were also checked. One researcher conducted the search, essays and data synthesis, as well as the subjectivity risk assessment, which was then reviewed by a second researcher. The inclusion criterion was association with interleukins relevant to MS pathogenesis and microglia cells. The exclusion criterion was lack of association with MS or interleukins in MS and duplication of information, included to date, as well as insufficient information on the subject of the review. Sources of funding were not considered in the search and selection of publications. 

## 3. Interleukin Characteristics

### 3.1. IL-1 

For two decades now, increased IL-1 production has been thought to be associated with MS being a disabling, inflammatory and neurodegenerative disease of the CNS. Through numerous clinical studies, the multifaceted role of IL-1 in MS pathophysiology has been demonstrated relatively recently [10]. However, it must be taken into account that the efficacy of currently available therapeutic agents that target the IL-1 system has not yet been satisfactorily demonstrated for MS, despite positive results from preclinical studies and successes in the treatment of other autoimmune diseases [11].

IL-1β, like IL-1α, is a precursor, inflammatory cytokine in the IL-1 group. As a result of binding to the IL-1 type 1 receptor (IL-1R1), many inflammatory reactions are triggered, which can activate the mechanisms of both innate and acquired immunity [10]. The cellular response is triggered by binding IL-1β to the transmembrane receptor IL-1R1 (or IL-1R3). Thanks to caspase-1 and inflammasomes, the precursor protein IL-1β is processed, resulting in its active form, secreted as a result of the appearance of an inflammatory signal [12]. IL-1β enhances inflammation in the course of autoimmune diseases, and also has the ability to stimulate the production of IL-6 [12,13].

Many of the recently published reports using animal models expose the important role of IL-1 not only towards the peripheral nervous system, as previously thought, but also towards the CNS [10,14]. Particular attention is paid to the effects of IL-1 against immune cells and within the CNS, leading to the development and exacerbation of MS. These mechanisms range from demyelinating processes to excitotoxicity or synaptopathy [12]. The latter is defined as a disorder, associated with developmental delay and intellectual disability, usually mild to moderate. It is thought to be caused by an imbalance between GABA-ergic and glutamatergic transmission, consequently leading to the destruction of synapses [11,15]. 

The discovery of IL-1’s potential for both immune and non-immune functions within the CNS has established the perception of IL-1 as one of the key mediators of neuroinflammation [14]. 

IL-1 does not exclusively play a deleterious inflammatory role. Under physiological conditions, specific low levels of IL-1 are important for brain health. Non-immune-related functions of IL-1 include control of sleep, appetite and thermoregulation. Neuroendocrine effects and the ability to neurogenesis are also reported [13]. 

In the case of the potential implementation of therapies affecting IL-1, the previously mentioned non-immunological functions of IL-1 should be considered in the treatment of both acute CNS conditions and chronic diseases with neurodegeneration [10,11,12]. Some recently published studies provide clear evidence of a link between IL-1 cytokines and MS pathogenesis. Elevated levels of IL-1β have been found in the blood and CSF of patients with this disease [16]. A correlation with MS incidence, course and progression of the disease has also been demonstrated [17]. IL-1β has been revealed to have the ability to trigger the demyelination process [18]. IL-1 can also potentially serve as a predictive marker for the relapsing–remitting multiple sclerosis (RRMS) phenotype, as elevated levels of IL-1β have been detected in CSF during remission [17]. Moreover, observations in some patients suggest a link between CSF and excitotoxic-type neurodegeneration [19,20]. 

It is noteworthy that IL-1β is often found in white matter, but its expression there is detected at relatively low levels and is associated with leukocyte infiltration [18,20]. In contrast, microglia cells and haematogenous macrophages migrating into the CNS are important producers of this cytokine [18,20]. Gene polymorphisms and alterations in IL-1 expression are definitely relevant to MS pathogenesis [16]. It is also noteworthy that some of the therapeutic approaches used for MS, such as steroid therapy, interferon beta (IFNβ) therapy or natalizumab, induce a decrease in IL-1β levels in the CSF while increasing IL-1 levels in the blood [10]. This observation could, in the near future, be a direction for research to determine the usefulness of IL-1β as a marker, monitoring the effectiveness of pharmacotherapy, as well as the degree of disease progression.

This paper, and several earlier ones, indicate that the influence of IL-1 on the occurrence and course of MS and EAE is primarily based on CNS neuropathology. Ongoing studies using chimeric models corroborated by clinical data support the hypothesis of a detrimental contribution of the IL-1 system in MS, but it appears that its effects are double-edged [10]. Some reports on the pleiotropic role of IL-1 in the CNS suggest that, despite its obvious proinflammatory functions, IL-1β may also participate in tissue repair and remyelination and have neuroprotective effects [11]. It should be pointed out that an interesting lead for the scientific world is the further search for a broader understanding of the beneficial role of IL-1, both in the context of neurodegenerative diseases and in stimulating memory or improving cognitive abilities. 

### 3.2. IL-6 

Increased levels of the inflammatory cytokine interleukin-6 (IL-6) are very often observed in autoimmune diseases and in inflammatory reactions in the organism [21]. Cells that produce IL-10 have a protective effect, while those that synthesize IL-6 most likely contribute to inflammation in MS [3,22]. IL-6 was first obtained in 1986, as a stimulatory factor for B2 lymphocytes. It is worth noting that, until recently, it was thought that T cells were crucial in the pathophysiology of MS. However, it has recently been established that B cells are also essential in the pathogenesis of this disease [1]. This interleukin’s functions include, inter alia, the activation of kinases and transcription factors through IL-6R/gp130 complexes, which, in turn, affect IL-6 activity [23].

IL-6 exhibits the ability to transmit a signal thanks to mIL-6R, which is a classic signalling pathway. An alternative is signal transmission via sIL-6R, which is referred to as the transsignalling pathway [22]. In both cases, IL-6 binds initially to the receptor and then, with the complicity of the CBD domains with gp130, causes diverse biological effects. Signalling of the classical type occurs primarily in hepatocytes and leukocytes, which leads to the promotion of anti-inflammatory responses [24]. The formation of these responses—both pro- and anti-inflammatory—is due to the hexameric complex of IL-6R and gp130. Through it, Janus kinase (JAK)28 is activated, which has the ability to activate three signalling pathways [22]. For MS studies, route 3 is important, in which phosphoinositol-3 kinase (PI3K) is activated, and thus the protein kinase B (PKB)/Akt pathway, in which JAK phosphorylates and activates PI3K. Its task is to phosphorylate selected phosphatidylinositides to the form of phosphatidylinositol-4,5-bisphosphate (PIP2), as well as phosphatidylinositol-3,4,5-trisphosphate (PIP3) [21]. The latter acts by phosphorylating and activating the GDP/Akt. The described PI3K/Akt pathway consequently leads to the activation of NF-κB.32 Activation of the STAT3, NF-κB and PKB/Akt pathways contributes to the development of diseases such as MS and other autoimmune diseases [22,23,24].

The profile of action of this cytokine has been found to be very diverse—it includes stimulation of hepatocytes, growth promotion of mouse plasmacytoma or human myeloma, and it also shows the ability to induce acute phase reactions [24]. In addition to the stimulatory actions mentioned above, IL-6 may also act as an inhibitor, such as in the inhibition of antiviral antibody responses after immunization with vesicular stomatitis virus [25]. A number of recently published studies suggest that circulating B lymphocytes in the peripheral blood of patients suffering from multiple sclerosis produce significantly more IL-6 (with concomitantly reduced levels of IL-10) than is the case in control patients. 

The activity of anti-inflammatory and pro-inflammatory cytokines plays a critical role in the course of MS [26]. It is currently believed that IL-6 is one of the most important cytokines, playing a major role particularly in the RRMS. Nevertheless, the role and mechanism of action of IL-6 in MS pathogenesis has not been fully understood so far [2,22]. Currently available findings suggest that inter-individual differences in the genes encoding IL-6 may exhibit an effect on the course of the inflammatory response in MS. 

IL-6 has been demonstrated to play a remarkable part in regulating the number of T helper (Th)17 cells, which produce IL-17, and regulatory T cells (Treg) [24]. It has been proven that excessive synthesis of IL-6 can trigger diseases, proceeding with autoimmunity, such as rheumatoid arthritis (RA) or indeed MS, where the action of Th17 cells is considered a key cause of disease onset [20]. Therefore, given the important role of IL-6 in regulating the balance between Treg and Th17 lymphocytes, it is conceivable that controlling IL-6 activity may be a suitable therapeutic strategy for MS patients. This important statement may bring an alternative treatment strategy for patients suffering from this disease. Therefore, it is definitely worth confirming them with further large-scale research.

### 3.3. IL-17

IL-17 is a molecule with pro-inflammatory effects that was initially referred to as CTLA-8 [8]. It was first identified and described in the second half of the nineteen nineties as a cytokine, produced by T lymphocytes. Clearly, these lymphocytes are present in significant amounts in the lesions of MS and EAE, particularly often in the early stages of these diseases and during relapses [27]. This cytokine is characterized by its ability to stimulate stromal cells (e.g., fibroblasts), as well as producing cytokines such as IL-6, IL-8 (CXCL8), granulocyte colony-stimulating factor (G-CSF), and prostaglandin E2 [8]. Through microglia cells, IL-6 and CXCL2 secretion is induced, while through astrocytes, IL-6, IL-1β and nitric oxide (NO) release is promoted, leading to increased inflammation as well as neutrophil recruitment [8,28]. IL-17 also acts by triggering the formation of reactive oxygen species by endothelial cells of the blood–brain barrier (BBB) and interferes with normal BBB function due to inhibition of tight junction protein expression and endothelial cell (EC) contractility [8,28].

IL-17 levels have been shown to be elevated in the blood and CNS of patients with MS [27]. However, the role of IL-17 in CNS inflammation remains ambiguous. Indeed, it has so far been established that its action is not essential for the development of classic active or adoptive transfer of EAE, but it does have an effect on disease exacerbation [8,27].

Studies on IL-17 have been able to confirm the hypothesis that the role of this cytokine is based on the early initiation of a T-lymphocyte-dependent inflammatory response [27]. This molecule clearly plays an important role in autoimmune diseases. A number of studies to date have shown elevated levels of IL-17 in the serum or tissues of MS patients, and it has also been established that IL-17 deficiency or blockade has a beneficial effect in EAE, but to varying degrees [8,28]. It should be noted that, through studies conducted on animal models, the biological action of IL-17, the signalling pathway of this cytokine, and its cellular sources have also been revealed.

### 3.4. IL-18 

IL-18 represents a group of pro-inflammatory cytokines. Its synthesis occurs in cells such as microglia, antigen-presenting cells and macrophages [29]. The induction of interferon gamma (IFN-γ) secretion by NK cells and T helper cells is one of the ways in which this cytokine operates, which is why it is also referred to asIFN-γ inducing factor [30]. The maturation and release of IL-18, like IL-1β, is mediated by caspases associated with inflammasomes. 

To date, a number of scientific papers have already suggested the importance of IL-18, whose increased serum levels have been linked to the occurrence of autoimmune diseases. These include inflammatory bowel disease, juvenile idiopathic arthritis and MS [29,30]. Numerous studies are now available indicating a clear link between increased IL-18 levels and the pathogenesis of MS [29,30,31,32,33,34,35,36,37,38]. 

In a study by Jahanbani-Ardakani et al., the aim was to assess IL-18 levels and the prevalence of three SNPs (single nucleotide polymorphisms) of IL-18 in the serum of MS patients from Iran [29]. Significantly elevated serum IL-18 levels were detected in MS patients in this study. However, there were no significant differences in IL-18 allele frequency between MS patients and controls, although a higher frequency of the AC rs1946518 SNP genotype was found. Also, another study by Chen et al. found significantly higher IL-18 levels in MS patients than in healthy controls [32]. Nicoletti et al. reported similar results and demonstrated significantly higher IL-18 levels in patients with RRMS in the acute phase of the disease, compared to patients with RRMS in the stable phase [29,36]. Furthermore, significantly higher levels of IL-18 were found among patients with secondary progressive MS compared to patients with RRMS in the acute or stable phase of the disease [29]. 

Losy and Niezgoda, in addition to demonstrating significantly elevated IL-18 levels in patients with RRMS, found that IL-18 is elevated in MS patients with active MRI lesions compared to patients without such lesions [36]. Interestingly, some studies, conducted in a group of Bulgarian patients with RRMS, did not show elevated serum IL-18 levels [37,38]. Significantly higher levels of IL-18 in cerebrospinal fluid (CSF) have been reported among patients with MS compared to healthy controls [39]. However, other studies have reported IL-18 levels being normal or even slightly reduced in patients with chronic progressive MS and the RRMS form, with no recordable levels of IL-18 in CSF in the control group [20]. 

The normal functioning of the CNS requires low levels of interleukins such as IL-1β or IL-18. Their deficiency can lead to CNS dysfunction, but their values, above the norm, sustained over a prolonged period of time, can induce toxic effects on the nervous system [14]. 

IL-1β and IL-18 are thought to be involved in nerve inflammation and are also involved in CNS diseases such as epilepsy and Alzheimer’s disease (AD) [29].

It seems remarkable, when looking for correlations concerning IL-18, that significantly elevated levels of this IL were observed in the serum and CSF of the patients studied [13,40,41]. In contrast, in a study of a Bulgarian patient sample population, IL-18 levels were found to be within the normal range or even reduced, which could indicate some ambiguity in the interpretation of the role played by IL-18 in MS [35,38,42]. However, these measurements would require further study to determine what factors might be responsible for these discrepancies. 

It seems interesting to note a significantly higher level of IL-18 in patients with RRMS in the acute phase of the disease, compared to patients with RRMS in the stable phase [34,37]. It is also interesting to obtain the result of significantly higher IL-18 levels among patients with SPMS compared to patients with RRMS in the acute or stable phase of the disease. Thus, in the future, IL-18 could possibly be used as a marker to differentiate the MS phenotype. 

The discovery of elevated IL-18 levels among patients with active MRI lesions in the course of MS also introduces an interesting perspective, offering hope for the use of this IL in the diagnosis of MS and the monitoring of disease progression [35]. 

### 3.5. IL-12 

IL-12, a proinflammatory cytokine was the first molecule of the Interleukin-12 family to be identified and described, thus giving rise to this group. This family also includes IL-23, IL-27 and IL-35, which have been shown to be important regulators of host immunity [43]. 

IL-12 represents a heterodimeric protein. It is composed of p35 and p40 subunits. It is produced by several types of haematopoietic cells, most notably antigen-presenting cells (APCs), i.e., dendritic cells and macrophages. High levels of IL-12 and Th1 are reported in clinical samples among MS patients [44]. The relevance to the pathogenesis of CNS autoimmune diseases is further exposed by the fact that IL-12 and Th1 cell levels are elevated in experimental autoimmune uveitis (EAU), an animal model of human uveitis and EAE [9]. Likewise, in an autoimmune connective tissue disease such as RA, studies have shown significant increases in IL-12 and Th1 levels in serum and synovial fluid. Elevated levels of these parameters also show a correlation with disease activity [9]. These observations provide evidence that IL-12-induced differentiation of Th1 cells may be associated with the development of organ-specific autoimmune diseases.

There are a number of indications for the potential therapeutic use of bioengineered or natural human IL-12 cytokines, whose single-chain subunits may also represent a new class of therapeutic molecules [43]. The creation of new molecules based on the use of members of IL-6-related cytokines could lead to additional agents that would expand the existing pool of IL-12/IL-6 therapeutics [45,46]. 

Thus, further research in this area is highly encouraged so that, in the near future, the knowledge of the action of this unique group of cytokines can be expanded with new findings—among others, those concerning the possibility of other combinations of α and β subunits, their biological activity, effects and potential use in the treatment of inflammatory CNS diseases, including MS. 

Interestingly, it was noted that mice deficient in the IL-12p40 subunit did not develop EAE, while those with IL-12p35 developed the disease [43]. This may indicate the deleterious effect of excessive levels of IL-12p40 in the development of autoimmune inflammation and suggest that IL-23 (IL-12p40/IL-23p19) rather than IL-12p40 may show a more important effect on the development of EAE [47]. 

IL-12p40 homodimer levels are elevated in patients with MS, and treatment with an anti-IL-12p40 monoclonal antibody has demonstrated an effective result for this neurological dysfunction, which may suggest a role in the pathogenesis of the disease [9]. Nevertheless, it should be mentioned that administration of this antibody in a subsequent clinical trial did not inhibit inflammation. 

### 3.6. IL-23 

The first report of IL-23 opened the way for the discovery of Th17 lymphocytes. IL-23 is considered to be one of the most important mediators of neuroinflammation in many autoimmune diseases, including MS [46]. However, the mechanism of action of IL-23 and how it contributes to the development of these diseases has so far been poorly understood [48]. Even the first insights suggested that IL-23, rather than the associated IL-12, is the key cytokine contributing to CNS autoimmune diseases. To date, this conjecture has been confirmed by a number of studies, thus identifying the role of IL-23 in various disease entities—inflammatory bowel disease, RA or psoriasis [49,50,51]. As previously mentioned for CNS diseases involving autoimmunity, the results of numerous papers demonstrate a central role for IL-23 in inflammatory brain diseases such as MS [45,52]. One piece of evidence in support of this thesis may be the finding that mice with insufficient expression of the IL-23 receptor (IL-23R), as well as transgenic models with impaired expression of the IL-23 subunits p19 or p40, were unable to develop EAE despite attempts to induce it. This demonstrates, in all likelihood, the non-negligible influence of this cytokine on the occurrence of EAE [48,49]. In addition, increased levels of IL-23 in serum and CSF are often found in MS patients [53]. A single nucleotide polymorphism of the p40 subunit, which increases p40 expression, has also been shown to be associated with earlier disease onset in some patients (with a younger age of onset) [52]. It is noteworthy that, in addition to CNS autoimmunity, recently published studies indicate the importance of IL-23 in the pathogenesis of carotid atherosclerosis, the pro-inflammatory effects that occur after ischaemia in stroke, as well as the association of IL-12/-23 with the onset of Alzheimer’s disease [34,35,36,37]. As well, it appears that IL-23 may lead to impaired host defence mechanisms in infectious brain diseases such as West Nile virus, leading to leukocyte accumulation in infected tissue during brain inflammation [54]. Nevertheless, the detailed function of IL-23 in the course of the aforementioned conditions has not been clearly defined to date. 

IL-23 is a member of the IL-12 family of cytokines and is composed of a p19 subunit and a common p40 subunit, which it shares with IL-12 [46]. This molecule is synthesized by APC cells [55,56]. IL-23 functions by stimulating αβ and γδ T cells, thereby differentiating Th17 lymphocytes, which are of considerable importance for chronic inflammation and MS pathogenesis [43]. Laboratory analyses often reveal the presence of Th17 cells in the lesions of MS patients [50]. In conclusion, the impact of IL-23 on neuroinflammatory diseases is beyond doubt, but the mechanisms by which IL-23 is able to modulate these conditions still need further elucidation. To further describe and explain the importance of IL-23 in CNS inflammation in the study by Nitsch et al., a transgenic mouse model (GF-IL23) was generated, possessing astrocyte-targeted expression of both IL-23 subunits, i.e., p19 and p40. This type of GF-IL23 mouse reveals a tendency to spontaneously develop an ataxic phenotype that leads to degeneration of cerebellar tissue. In turn, inflammatory infiltrates were most prominent in both the perivascular and subarachnoid spaces [46]. The leukocytic infiltrates were clearly dominated by B lymphocytes. To investigate more precisely the effect of CNS-specific IL-23 synthesis on the overall inflammatory stimulus, it was decided to administer LPS (LPS-induced endotoxemia) to mice. This action resulted in an early and pronounced activation of microglia, increased cytokine production and, in contrast to control animals, the formation of lymphocytic infiltrates [48]. This model probably confirms the key role of IL-23 in the induction of inflammation in the CNS. This occurs in particular by promoting the accumulation of lymphocytes in and around the brain. Thus, the CNS-specific synthesis of IL-23 induces a cascade of inflammatory cytokines, leading to the activation of microglia, astrocytes and, ultimately, tissue damage [50].

### 3.7. IL-4

As a pleiotropic cytokine, IL-4 is known to play an important role in type 2 immune responses. It is produced by Th2 cells, while it exerts its effects by inhibiting Th1 cells [57]. The progression of MS has been shown to be inhibited by cytokines that are produced by Th2, one of which is IL-4. Using the EAE model as an example, it has been observed that the use of IL-4 has a mitigating and protective effect [35]. An increase in the number of PBMN (peripheral blood mononuclear cells—cytotoxic T cells, helper T cells, NK cells and B cells), which secrete IL-4, has been reported in both the RRMS and secondary progressive MS (SPMS) [57]. Given that IFN-γ production is inhibited specifically by IL-4-producing cells, the increase in IL-4-secreting cells in MS may represent a marker for cells attempting to counteract Th1-associated tissue neurodegeneration in MS. The study by Calabresi et al. reported that cells in the cerebrospinal fluid of MS patients predominantly expressed TNF-α, but IL-4 expression was not present. The opposite observation was obtained for two patients in remission [40]. These authors suggested that the absence of IL-4 expression in all except the most stable patients supports the hypothesis that IL-4 may be a regulatory cytokine in MS, which is consistent with the data obtained in the EAE model. 

The study authored by Windhagen et al. also found that patients with SPMS, in the presence of IL-12 or anti-IL-4 antibodies, tended to have fewer IL-4-secreting T cells compared to controls or patients with the RRMS form [58]. This suggests a defect in IL-4 production in MS patients and may support the notion that there are correlations between IL-4 production and MS phenotype. 

In regard to the therapeutic effect of IL-4 on the course of MS, G. Casella et al. showed that IL-4 administration significantly modulated neuroinflammation and also significantly reduced clinical symptoms in experimental autoimmune encephalitis, spinal myelitis and a mouse model of MS. It has also been demonstrated in primate models of EAE that delivery of the cytokine IL-4 to the CNS during gene therapy produces a very effective result in the treatment of ongoing nerve inflammation [35]. 

Noteworthy is a study that evaluated the in vivo immunomodulatory effect of single and combined treatment with all-trans-retinoic acid (ATRA) and docosahexaenoic acid (DHA), on peripheral blood mononuclear cells (PBMC) in patients with RRMS who were also receiving interferon beta (IFN-β) [47]. 

In this study, ATRA (5 nM) was shown to affect IL-4 gene expression in human T cells, while also leading to changes of these cells to a Th2 form [47]. However, the results of the referenced study did not reveal a significant effect on IL-4 gene expression with either combination or single therapy using DHA and ATRA. It is worth mentioning that inconsistent results for IL-4 expression have already been reported in previously published papers. In a study by Yang et al. it was reported that administration of ATRA to mice could significantly reduce IL-2 and IL-4 gene expression, and, moreover, this suppressive effect of ATRA on IL-4 was achieved even at the lowest concentration used (5 ng/mL) [47]. It has been speculated that IL-2 signalling plays a key role in ATRA-mediated activity, while the significant decrease in IL-4 may be due to IL-2 suppression. However, in another investigation that was performed on a human T-cell model by Parra et al., no significant changes in IL-4 and IL-10 expression levels were observed after treatment with ATRA. 

### 3.8. IL-10 

IL-10 is most synthesized by lymphocytes and myeloid cells. Some other cells, during active inflammation, also show the ability to produce it [6,59]. IL-10 has the ability to inhibit many cytokines, such as IL-1β, IL-6, IL-12, IL-18, GM-CSF and TNFα. Its effects include increasing the production of other anti-inflammatory mediators, including IL-1β receptor antagonist [2,60]. 

There are reports showing that IL-10 has the ability to inhibit the activity of antigen-presenting proteins, such as CD80, CD86, IL-12, etc. In turn, in the presence of other cytokines, IL-10 tends to increase the proliferation and differentiation of human B lymphocytes into immunoglobulin-secreting cells. It is noteworthy that the expression and role of IL-6 and IL-10 can be controlled by many factors, which include other cytokines, genetic polymorphisms or non-coding RNAs [9,10,11,13,15]. The anti-inflammatory role of IL-10 has been proven in disease entities such as diabetes, inflammatory bowel disease, the previously mentioned RA and MS [61]. 

One form of treatment for MS patients used to date, which has been associated with simultaneous upregulation of IL-6 and IL-10, is therapy with glatiramer acetate (GA), a myelin basic protein (MBP) analogue approved by the Food Drug Association (FDA) [60,61]. 

Glatiramer acetate has been shown to increase IL-10 production [59], with isolated forms of GA-specific T cells secreting IL-6 [60]. The role of IL-6 in GA-mediated therapy has not been fully determined to date. This interleukin has a supportive effect on Th2 differentiation in T lymphocytes as a result of inducing IL-4 expression, and in mouse models, it acts by blocking IFN-γ signalling [61,62]. These findings make GA a promising research target for the dual role of IL-6 in both Th2 and Th17 responses, which may be both beneficial and detrimental in MS. Further work would need to be planned to determine whether IL-10 has EAE-like effects in human patients, which could provide new therapeutic benefits.

Reports from Egyptian researchers appear to be remarkable. The work by Sedeeq et al. aimed to evaluate IL-10 levels and determine its impact on the nature and course of MS in a study group of patients [63]. Approximately one-third of this group was characterized by an EDSS (Expanded Disability Scale Status) score ranging between 4–6 in the remission phase, while the disease duration was set at a maximum of 13 months, suggesting an aggressive disease course in many of these subjects. It was also noted that the predominant symptoms in almost half of the study population were motor and cerebellar deficits, further highlighting the poor prognosis of MS in this group [63]. We found that IL-10 was determined to be higher in RRMS patients in the remission phase than during the relapse phase, which is consistent with many previous findings revealing a reduction in IL-10 levels in the relapse phase relative to remission [59]. The demonstration of an increase in IL-10-producing cells after the end of the first year of treatment with IFN-β also seems to be an important finding [60]. These phenomena may be explained by the fact that IL-10 participates in the development of a pattern of anti-inflammatory cytokines, which is achieved through inhibition of the synthesis of numerous Th1 cell-related cytokines. Consequently, IL-10 thus contributes to the remission phase of the disease [59]. 

In contrast, for miR-96, the results showed that its expression was higher in RRMS patients in remission than in relapse. Expression was also higher among control patients than in patients with RRMS in relapse [63]. The results for miRNA-96 in MS are few and novel. They also do not differ from previous reports from the Middle East and Northern Africa (MENA) region, which may indicate that, like IL-10, it is a relevant parameter [61,62]. At the same time, this opens the way for further studies on this topic. Previous work investigating CNS changes and blood cell profiles in patients with MS has established that miRNA expression is up- or down-regulated in MS [64]. This study attempted to detect a potential correlation between elevated IL-10 levels and higher miR-96 expression, both during relapse and in remission [63]. Nevertheless, no such correlation was found. This finding could provide an interesting direction for further exploration among other patient populations to determine whether there are any correlations between biomarkers such as IL-10 and miR-96. If this is not found, it would be necessary to determine through which anti-inflammatory pathway miR-96 is involved. 

### 3.9. IL-13 

IL-13 is an anti-inflammatory cytokine synthesized primarily by Th2 cells. To date, it has been shown to have a significant effect in various inflammatory diseases by binding to receptors such as IL-13R, IL13-Rα1 and IL-13Rα2 [65]. To date, evidence of IL-13’s ability to relieve clinical symptoms and reduce inflammatory cell infiltration in a model of EAE has already been demonstrated. 

A study by M. Azimzadeh et al. investigated the immunoregulatory and neuroprotective effects of human adipose tissue-derived stem cells (hADSCs) overexpressing IL-11 and IL-13 in mice with EAE [65]. The results of this work showed that clinical signs and mononuclear cell infiltration into the spinal cord of EAE mice (such infiltration indicates the presence of chronic inflammation) were significantly less severe in mice treated with IL-11 and 13-hADSCs [55,56,65]. 

It was observed that oligodendrogenesis and remyelination processes occurred more efficiently in the aforementioned treated group, compared to the untreated EAE group. In neurodegenerative lesions, oligodendrocyte reconstitution probably plays an important role in myelin reconstitution [55,65]. In the cited study, it was shown that neurological dysfunction, demyelination and inflammation in EAE mice were significantly reduced by intravenous administration of hADSCs overexpressing IL-11 and 13 [47,65]. 

These insights may highlight the role of oligodendrogenesis in IL-13-treated mice, and may also suggest that IL-13, together with its neuroprotective effect of hADSCs, is a factor that activates the development of oligodendrocyte lineage in damaged areas of the CNS. 

It is also worth mentioning that smoking worsens the prognosis of MS. A study by J. Correale et al. provided evidence that smoking in patients with MS leads to increased production of IL-13 and IL-6, which has been associated with reduced indoleamine 2,3-dioxygenase (IDO) activity [56,66,67]. IDO is an enzyme that is one of the main anti-inflammatory and immunosuppressive factors. Its role is to maintain immune tolerance, participate in the catabolism of tryptophan to kynurenine, or inhibit the activity of T lymphocytes and NK cells [66,67]. It also possesses the ability to enhance angiogenesis and Treg lymphocyte synthesis [56]. Therefore, it is now accepted to be one of the most important enzymes involved in tissue regeneration and the mitigation of inflammation. IDO has been shown to have a significant role after organ transplantation and in autoimmune diseases. It also has been found to influence the course and prognosis of EAE [66,67]. 

As a growing literature also provides considerable information on the harmfulness of electronic cigarettes, it is reasonable to speculate that a comparable negative influence may also be occurring with the use of these vaping devices [6,7].

Of particular note in the analysis of published studies on IL-13 is the protective effect of hADSCs. These cells, which originate from connective tissue, cause the alleviation of clinical symptoms in EAE [65,68]. Capable also of enhancing oligodendrogenesis and myelin reconstitution, these cells seem to represent an interesting target for further research. In our study, we would also like to highlight the results presented by Guglielmetti et al. [64]. These authors used cuprizone (a copper chelator) in a model of EAE to induce intense inflammatory demyelinating lesions within the white matter by injecting it intravenously into mice [68,69,70]. This action resulted in the induction of markers adequate for alternative activation in both macrophages and microglia cells. Microglia have been shown to have an effect in oligodendrocyte survival and, moreover, the observed myelin degeneration was much milder during its activity [70,71]. The next step needed would be to attempt a similar investigation in a human model of MS, as well as other studies to assess the effect of IL-13 administration in stroke, AD and other diseases with neurodegeneration. 

### 3.10. IL-3 

Despite research on IL-3, relatively little is still known about the role of this cytokine in the pathogenesis of MS and EAE in humans. IL-3 is a member of the haematopoietic cytokine family, having short α-helices [72]. This family also includes granulocyte-macrophage colony-stimulating factor (GM-CSF) and IL-5 [69]. Although the role of GM-CSF in EAE has been well understood and described, limited information is known about the importance of IL-3. The few reports to date describe the effect of IL-3 overexpression on the demyelination process and on neurotoxicity [73]. Although the functions performed by IL-3 and GM-CSF are very similar, it is the second molecule that is thought to exacerbate the course of EAE, which is explained, among other things, by monocyte activation [74]. The effects of IL-3 include many cell types. It involves either activating cells or promoting their survival, the most common of which are monocytes, DCs, basophils, mast cells, B lymphocytes and endothelial cells [75]. The clear involvement of individual leukocyte types in the development and progression of EAE has been demonstrated. Only the effect of mast cells in this regard remains uncertain [76]. Activated CD4+ T lymphocytes are indicated as the primary source of IL-3 secretion in EAE. Nevertheless, this cytokine can also be produced by B lymphocytes, basophils, neurons or microglia cells [77]. 

The work by Renner et al. aimed to analyse the role of IL-3 in EAE, which was performed using peptide-induced myelin oligodendrocyte glycoprotein (MOG) [72]. For this purpose, mice were injected with a blocking monoclonal antibody against IL-3. Mice with genetic IL-3 deficiency and individuals after injection of recombinant mouse IL-3 were also evaluated [72]. In this study, CD4+ lymphocytes were identified as one of the superior producers of IL-3. It is worth mentioning that IL-3 release after restimulation with MOG peptide 35–55 in the spleen is dependent on the presence of CD4+ T lymphocytes, whereas in the CNS, IL-3 expression shows a close correlation with the influx of CD4+ T lymphocytes [78]. This study also revealed a correlation of cerebral IL-3 expression with T-lymphocyte migration into the brain. Upon immunization with MOG, IL-3 expression in the CNS shows a significant increase at day 14, while it decreases to baseline at day 20, as does T-lymphocyte infiltration [77]. In astrocytes, overexpression of IL-3 results in primary demyelination due to microglia activity. With the onset of white matter lesions, motor impairment occurs [79]. It has also been shown that the administration of recombinant EAE to mice exacerbates clinical signs and the neuroinflammatory process, while the use of IL-3 blockade by administration of a monoclonal antibody attenuates the course of EAE [80]. Similar insights have been made in mice with a genetic deficiency of this cytokine [81]. Furthermore, it is noteworthy that among patients with RRMS, IL-3 expression was found to be significantly increased as a result of T-cell activity during disease relapses [72]. 

Increased IL-3 expression has been reported not only in mice with EAE, but also among humans with MS. IL-3 has been observed to be present in the PMR during the course of this disease and has been shown to correlate with both diagnosis and disease severity [56]. Transcriptional analysis of cytokine expression in brain samples from MS patients showed an increase in IL-3 expression compared to controls [82].

The undisputed neuroprotective effect of IL-3 in AD has been demonstrated by initiating the elimination of amyloid beta (Aβ) and tau protein through microglia cells [70]. However, the relevance of IL-3 to other neuroinflammation-related disorders, particularly MS or EAE, still remains a mystery. Undoubtedly, in the above disease entities, the effect of this cytokine is not beneficial, but most likely detrimental, contributing to the development of the inflammatory process [70,75]. In the currently available scientific literature, it is possible to find information on the EAE model, according to which IL-3 most likely plays a role in neuronal demyelination [72,74,80]. Nevertheless, these reports report inconclusive results. In particular, the cell types responding to this cytokine in the CNS remain undetermined for IL-3. The only mechanisms of action confirmed so far, with which novel therapeutic approaches can be hoped to be developed, concern the cell communication pathways between resident glial cells and infiltrating immune cells in neuroinflammation [76]. It has been demonstrated that both resident astrocytes and infiltrating CD44 and CD4+ effector T cells have the ability to produce IL-3 in inflammation within the CNS [75]. 

IL-3 has also been shown to affect both microglia and myeloid cells of peripheral origin as a result of IL-3Rα expression [75,83]. An interesting conclusion was reached by the authors of the paper by Kiss et al. [75]. Indeed, it was found that in human MS and EAE, astrocytes located within the CNS, together with infiltrating CD44hiCD4+ T lymphocytes, synthesize IL-3. This, in turn, initiates a migratory and chemotactic programme in collaboration with microglia cells acting in the CNS with IL-3Rα expression. IL-4 manages these processes with the participation of peripheral myeloid cells to consolidate immune cell recruitment and the CNS inflammatory process [75,84]. This pathway of action is associated with the exacerbation of clinical symptoms of MS and EAE. It has been shown that, in humans, measurement of IL-3 in CSF can be used as a predictor of diagnosis and also serve to determine the severity of MS exacerbation [76,80]. 

In mice, the deletion of Il3 or Il3rα systemically alleviated EAE and reduced neuronal demyelination. It also reduced recruitment of immune cells to the CNS as a result of reducing chemotactic signals originating in myeloid cells [75]. Therefore, in the near future, an extensive search should be implemented to resolve the meaning of the relationship between IL-3 and IL-3Rα. This would certainly broaden knowledge of the pathophysiology of MS and perhaps provide the opportunity to develop alternative therapies for CNS inflammatory diseases. 

The characteristics and possibilities for the diagnostic and therapeutic use of the interleukins discussed in this review are included in Table 1.

## 4. Discussion 

Until recently, the molecules called interleukins were quite a mystery. The scientific world has known about this subgroup of cytokines for a relatively short time, with some of the first reports of them in research dating back to the 1970s [50]. As individual interleukins were systematically discovered, the mechanisms of action and the role they play in the pathogenesis of numerous diseases, especially those involving autoimmunity or progressive inflammation, remained unclear [3,4]. Originally, they were identified as agents operating within the leukocyte population, but it is now known that they may also be produced by other cell groups, such as fibroblasts, among others [51]. Currently, it can be assumed that of the interleukins, IL-6 is the most extensively studied and described, also in relation to its significance for neurodegenerative diseases, including AD and MS [52]. 

It seems remarkable to find the possibility of an effect of IL-1 on improving memory and cognitive abilities, a phenomenon referred to as synaptic plasticity [85]. Currently, this phenomenon is attempted to be explained by the expression of IL-1β within the hippocampus, which is induced by the learning process. This expression promotes the maintenance of long-term synaptic potentiation (LTP) [86]. Observations show that small, short-term increases in IL-1β levels result in memory enhancement, but any increase or decrease in the expression of this cytokine beyond the reference range leads to the occurrence of hippocampal-dependent memory deficits [10,86]. Similarly, IL-1 has been shown to have beneficial effects on neuronal survival through the regulation of neurotrophic factors and oligodendrocyte differentiation (OLG) [13,87]. 

It is notable that regular, moderate physical activity has a proven beneficial therapeutic effect on patients suffering from MS. Exercise has been shown to affect changes in measurements of peripheral cytokine levels. Kouvelioti et al. reported significantly higher levels of IL-6, IL-1β and TNF-α in patients who were prescribed cycling and high-intensity interval running [88]. In this context, the findings in the paper by Shobeiri et al. appear to be of particular relevance. In their meta-analysis, results concerning the effects of systematic exercise on both plasma and serum levels of selected interleukins were reviewed, with particular attention given to IL-6 and TNF-α [89]. Factors exerting an undeniable influence on the differences in cytokine levels after exercise are the gender of the patients and their age [53]. A study has shown that exercise decreases IL-6 production by the CNS, leading to inhibition of microglia cell activity. Furthermore, IL-6 produced by muscle tissue during exercise promotes the activity of anti-inflammatory cytokines, including IL-10, while reducing the pro-inflammatory effects of TNF-α [45,53]. 

However, this is not the only beneficial mechanism, as a reduction in IL-6 may lead to an improvement in the elimination of pathogens or nullify T-cell activation, which is known to have the ability to alleviate the course and clinical symptoms of MS [45]. Previous work has also demonstrated a link between physical activity and interleukin levels in patients suffering from other diseases with neurodegeneration, including Huntington’s chorea and AD [90,91]. Consideration is also given to the activity of other cytokines, such as IL-17, as well as the fact that measurements are not always conclusive [91]. Further studies are certainly needed to confirm the conclusions reached to date or to formulate further ones, thereby shedding new light on the therapeutic approaches discussed. 

The significant characteristic of the IL-12 family of cytokines, which in a way opens the possibility of different ways of participating in host immunity, is the presence of a common promiscuous chain [43]. Elements of the IL-12 protein structure consisting of a single chain allow different immune cells to recognize similar cytokine signals, inducing immune tolerance or, on the contrary, immune suppression [9]. It should be taken into account that the individual IL-12 α or β subunits also exhibit intrinsic biological activities that are not necessarily similar to their heterodimeric partners [92]. Consequently, this may sometimes limit the beneficial effect of the heterodimeric IL-12 variant. 

However, the IL-12p40 subunit is also produced independently, being a homodimer [93]. To date, there has been no analysis of p40 homodimer levels in human serum, but it has been demonstrated emphatically in the EAE model. It is noteworthy that the IL-12p40 homodimer binds the IL-12 receptor and has so far only been linked to a function opposing IL-12 activity [43,94]. IL-12p40 homodimer levels reach elevated levels in patients with MS, and anti-IL-12p40 treatment has a neuroprotective effect in this condition [72].

It is notable that IL-4 has been shown to have mitigating and protective effects on the CNS, as well as inhibitory effects on the development of MS, as demonstrated in a mouse model of EAE [57,60]. An increase in the number of PBMN (peripheral blood mononuclear cells—cytotoxic T cells, helper T cells, NK cells and B cells) secreting IL-4 in both RRMS and SPMS has previously been found [34]. An inhibitory effect of IL-4-producing Th2 cells (also IL-10 or IL-13) on IFN-γ, among others, has been observed. As a result, an increase in the number of IL-4-secreting cells in MS may be a promising marker for the promotion of cells attempting to suppress Th1-associated tissue neurodegeneration in MS [60,95]. This observation may serve to potentially use IL-4 as a diagnostic marker or develop new therapeutic approaches using specifically IL-4 and Th2 cells. Nevertheless, further extensive research in this direction is needed. 

Despite numerous studies conducted to date, the mechanisms of action of IL-3 in CNS disorders remain insufficiently investigated. The same can be concluded about the potential therapeutic opportunities that this cytokine could bring [72,75].

## 5. Conclusions and Perspectives

It can be clearly indicated that ILs have immunoregulatory effects. Some of these molecules show a neuroprotective inhibitory effect on the inflammatory process, while others exacerbate the disease process.

It should be emphasized that further studies need to be planned, the main focus of which, in the case of IL-1, would be to clarify the efficacy of currently available therapeutic agents for use in MS, whose action is based on this cytokine. The described non-immunological functions of IL-1, which appear promising for the treatment of neurodegenerative diseases, including MS, should also be further investigated. In the context of the therapeutic approaches already in use in patients suffering from MS, an equally promising line of research seems to be the possibility of determining the usefulness of IL-1β as a marker, monitoring the effectiveness of pharmacotherapy, as well as the severity of the disease. 

Further research could shed new light on this IL in the context of its pleiotropic effects—the neuroprotection it exhibits and its participation in tissue repair or remyelination. Additionally, its ability to improve memory or cognitive abilities seems to be of interest. 

It should be emphasized that it is reasonable to implement an attempt to confirm the role of IL-6 in regulating the balance between Treg and Th17 lymphocytes, as it may represent a breakthrough therapeutic strategy. 

It would also be necessary to confirm whether indeed IL-17 deficiency or blockade has a positive effect on EAE, and whether this is analogous for MS in humans, and to what extent. 

Looking through the prism of the inconclusive results, regarding IL-18, further attempts to demonstrate the correlation of its elevated levels with the occurrence of the acute phase of the disease, as well as to determine whether it is indeed higher in patients with SPMS than RRMS, and how these results are influenced by belonging to different ethnic groups, are indispensable. We suggest that it may be possible to use this IL as a potential marker to differentiate between forms of MS and to monitor disease progression. The discovery of elevated IL-18 levels in patients with active MRI lesions in the course of MS also introduces an interesting perspective, offering the prospect of using this IL in MS diagnosis and monitoring disease progression.

It is also suggested that single-chain subunits of the cytokine IL-12 could give rise to a new type of molecule with therapeutic activity, alternative to those currently in use. Significant research in this direction is definitely needed.

In the context of IL-10, further work would need to be undertaken to determine whether IL-10 shows analogous therapeutic benefits to EAE. Undeniably important for this molecule seems to be the further identification of a correlation between the markers miR-96 and IL-10, for a population other than Egyptian, as in this population, a significantly statistical correlation has not been demonstrated. 

These findings make GA a promising research target for the dual role of IL-6 in both Th2 and Th17 responses, which may be both beneficial and detrimental in MS.

Offering hope for a new therapeutic modality appears to be hADSC, which acts as a neuroprotective agent, reducing demyelination and inflammation in mice—but its effects need to be investigated in human MS patients. In the near future, it is also worthwhile to further clarify what the detrimental effects of vaporization devices are in the context of IL-13.

These insights may highlight the role of oligodendrogenesis in light-treated mice, and may also suggest that IL-13, together with its neuroprotective effect of hADSCs, is a factor that activates the development of oligodendrocyte lineages in damaged areas of the CNS. 

Finally, an important avenue for further research is to evaluate the effect of administering both IL-13 and IL-3 on the course and prognosis of MS, in order to compare the effects obtained for the EAE model.

Undoubtedly, ILs have great potential for use in both predictive, diagnostic and therapeutic areas, and as such, the maximum possible depth of research into this matter is essential.

## Figures and Tables

**Table 1:** Characteristics and possibilities of diagnostic and therapeutic use of the described interleukins.

	Mechanism of Action	Character (Pro-Inflammatory/Anti-Inflammatory/Other)	Diagnostic and Therapeutic Possibilities
IL-1β	Ability to initiate the process of demyelination; effects against immune cells and within the CNS, leading to the development and exacerbation of MS; demyelinating, excitotoxicity and synaptopathy; immune functions within the CNS; Under physiological conditions, specific, low levels of IL-1 are important for brain health; Neuroendocrine action and neurogenesis ability.	Anti-inflammatory, pleiotropic	IL-1 could potentially serve as a predictive marker for the RRMS phenotype, as related to the finding of its elevated levels in CSF during remission; prediction of the occurrence of excitotoxic neurodegeneration; prediction of the occurrence of leukocyte infiltration in white matter.
IL-6	Activation of kinases and transcription factors via IL-6R/gp130 complexes; stimulation of hepatocytes, stimulation of growth of mouse plasmacytoma or human myeloma, ability to induce acute phase responses; IL-6 can act as an inhibitor, for example, when inhibiting antiviral antibody responses after vesicular stomatitis virus immunization; regulation of the number of IL-17-producing Th17 cells.	pro-inflammatory	Given the important role of IL-6 in regulating the balance between Treg and Th17 lymphocytes, it is conceivable that controlling IL-6 activity may be a suitable therapeutic strategy for patients with MS.
IL-10	The ability to inhibit multiple cytokines: IL-1β, IL-6, IL-12, IL-18, GM-CSF and TNFα; IL-10’s actions include increasing the production of other anti-inflammatory mediators, including the IL-1β receptor antagonist. It is likely that IL-10 has the ability to inhibit the activity of antigen-presenting proteins, e.g., CD80, CD86, IL-12, etc. The expression and role of IL-6 and IL-10 may be controlled by many factors, which include other cytokines, genetic polymorphisms or non-coding RNAs.	anti-inflammatory	IL-10 may serve as a predictive marker of the course of MS.
IL-18	IL-18 and IL-1β deficits can lead to CNS dysfunction, but values, above the norm, sustained over a long period of time, can have toxic effects on the nervous system; IL-18 and IL-1β are most likely involved in nerve inflammation and are also involved in the course of CNS diseases such as epilepsy and Alzheimer’s disease.	pro-inflammatory	IL-18 may serve as a predictive marker of the course of MS.
IL-4	Playing an important role in the type 2 immune response; using the EAE model as an example, it has been observed that the use of IL-4 shows a mitigating and protective effect; In both RRMS and the secondary progressive form of MS (SPMS), there was an increase in the number of PBMN (peripheral blood mononuclear cells—cytotoxic T cells, helper T cells, NK cells and B cells) that secrete IL-4; Likely a regulatory cytokine in MS.	pleiotropic	Because IFN-γ production is inhibited by IL-4-producing cells, the increase in IL-4-secreting cells in MS may be a marker for cells attempting to nullify Th1-associated tissue neurodegeneration in MS; increase in IL-4-secreting cells in MS may be a marker for cells attempting to abrogate Th1-associated tissue neurodegeneration in MS; the defect in IL-4 production in MS patients may support the notion that there are correlations between IL-4 production and the MS phenotype; in the context of the therapeutic effect of IL-4 on the course of MS, it has also been demonstrated in primate models of EAE that delivery of the cytokine IL-4 to the CNS during gene therapy produces a very effective result in treating ongoing neuroinflammation.
IL-13	Mitigation of clinical symptoms and reduction in inflammatory cell infiltration in the EAE model; Oligodendrogenesis and remyelination processes occur more efficiently compared to the untreated EAE group.	anti-inflammatory	it has been shown that neurological dysfunction, demyelination and inflammation in EAE mice were significantly alleviated by intravenous administration of hADSCs overexpressing IL-11 and 13; IL-13, along with its neuroprotective effects of hADSC, is a factor that activates the development of oligodendrocyte lineages in damaged areas.
IL-12	IL-12 has been shown to be crucial for Th1 differentiation; mode of action in MS pathogenesis not fully understood; relevant to MS pathogenesis is shown by the fact that IL-12 and Th1 cell levels are markedly elevated in EAE; mice deficient in the IL-12p40 subunit did not develop EAE, while those with IL-12p35 developed the disease, which may indicate the deleterious effect of excessive levels of IL-12p40 in the development of EAE; most likely, it is IL-23 (IL-12p40/IL-23p19), rather than IL-12p40 that shows a more important effect on the development of EAE.	pro-inflammatory	IL-12p40 homodimer levels are elevated in patients with MS, and treatment with an anti-IL-12p40 monoclonal antibody showed an effective effect for this neurological dysfunction (while its administration in a subsequent clinical trial did not inhibit inflammation), suggesting a role in the pathogenesis of the disease.
IL-23	Mode of action not fully understood; results of various works prove the main role of IL-23 in inflammatory brain diseases such as MS; increased levels of IL-23 in serum and cerebrospinal fluid are often found among patients-people with MS; marked accumulation of B lymphocytes indicates a role for IL-23 in attracting or accumulating B cells into the subarachnoid space and into the CNS parenchyma, a well-described pathological feature of MS and a central role for B cells in the pathogenesis of MS	pro-inflammatory	Positive effects of B-lymphocyte-depleting therapies on the course of the disease have been confirmed; Potential use of anti-IL-23 therapy to enhance the above effect.
IL-3	Induction of activation or increased survival of cells, among which monocytes, DCs, basophils, mast cells, B lymphocytes and endothelial cells are most commonly mentioned; still relatively little is known about the role of IL-3 in human MS and EAE.	It does not show an effect on inflammatory processes, but on haematopoiesis.	IL-3 is present in human PMR and shows a co-relation with both the diagnosis and severity of the disease; data indicate that IL-3 plays an important role in EAE and may represent a new target for the treatment of MS; injection of recombinant IL-3 exacerbates EAE symptoms and brain inflammation; in patients with RRMS, IL-3 expression by T cells is significantly increased during relapsing-remitting episodes.
IL-17	Ability to stimulate stromal cells (e.g., fibroblasts), as well as production of cytokines, or prostaglandin E2; Enhancement of inflammation, recruitment of neutrophils; Early initiation of inflammatory response, dependent on T lymphocytes; acting by triggering the formation of reactive oxygen species by endothelial cells of the blood–brain barrier (BBB); the role of IL-17 in inflammation of the nervous system remains; its action is not necessary for the development of classical active or adoptive transfer of EAE, but it exerts an effect on the exacerbation of the disease.	pro-inflammatory	Many previous works have shown elevated levels of IL-17 in the serum or tissues of patients with MS; it has been established that IL-17 deficiency or blockade has a beneficial effect in EAE.
